# Comment on ‘Lean, Non‐Autoimmune Young‐Onset Diabetes in Bangladesh: A Metabolically Obese Phenotype With Disproportionate Insulin Secretory Defect’

**DOI:** 10.1002/edm2.70373

**Published:** 2026-10-22

**Authors:** Wadivkar Pradnayshree, Bahendeka Silver, Kibirige Davis

**Affiliations:** ^1^ Department of Endocrinology Global Diabetes Institute, Albert Einstein College of Medicine New York New York USA; ^2^ Department of Medicine Uganda Martyrs University School of Medicine Kampala Uganda; ^3^ Department of Medicine Uganda Martyrs Hospital Lubaga Kampala Uganda; ^4^ Medical Research Council/Uganda Virus Research Institute and London School of Hygiene and Tropical Medicine Uganda Research Unit, Non‐Communicable Diseases Program Entebbe Uganda

To the Editor,

We read with great interest the article by Hasan et al., which evaluated the metabolic profile of lean non‐autoimmune young‐onset diabetes in Bangladesh [1]. We commend the authors for evaluating an important but neglected diabetes phenotype among low‐ and normal‐BMI young individuals, potentially affecting millions of individuals in low‐income settings. We appreciate that the authors narrowed down the phenotype by excluding participants with pancreatic pathology, monogenic variants and fasting C‐peptide levels below 200 pmol/L.

The study's ‘lean’ category in the main text combines participants with BMI < 18.5 kg/m^2^ and those with BMI 18.5–22.9 kg/m^2^. Given that the authors concluded that these ‘lean’ subjects have modest insulin resistance and metabolic dysfunction, we looked with interest at the BMI‐based sub‐analysis (in the supplementary material) and at whether the leanest subjects were insulin‐resistant. Of the 53 lean DM participants, only 9 had BMI < 18.5 kg/m^2^. The authors reported that low‐ and normal‐BMI DM participants showed similar phenotypic and metabolic features. However, we wanted to highlight that the waist‐hip ratio, waist circumference and waist‐height ratio (*p* = 0.033) were lower in the low‐BMI DM group than in the normal‐BMI DM group. Additionally, median triglycerides were much lower (within the normal range and closely matching the median for the lean non‐DM group) in the low‐BMI DM subgroup than in the normal‐BMI subgroup. Median HOMA2‐IR, fasting C‐peptide and fasting insulin were also lower in the low‐BMI DM group than in the normal‐BMI group. We recognize that none of these differences were significant except for the waist‐height ratio. However, the direction and magnitude of the differences are consistent across nearly every relevant variable. The low‐BMI DM subgroup appears consistent with the recently classified Type 5 Diabetes (T5D) phenotype cited in this paper, and we believe repeat laboratory and statistical measurements would help clearly distinguish it as a distinct phenotype.

Regarding lipid data in the article by Hasan et al., measurements were obtained cross‐sectionally from participants with severe, uncontrolled hyperglycemia, without prior correction of glucotoxicity, as mentioned by the authors. The median HbA1c in the lean DM subgroup was 11.6%, which may have contributed to the apparent dyslipidemia in this group. The same caution applies to HOMA2‐IR, since insulin resistance estimated during severe hyperglycemia is inflated by glucotoxicity and does not necessarily reflect participants' baseline state [2]. Therefore, the glucose, lipid and HOMA‐IR values used by the authors to identify ‘metabolic dysfunction’ in the lean DM group may, in fact, primarily reflect glucotoxicity. Therefore, we propose that repeating these measurements after correcting for glucotoxicity would help assess insulin sensitivity in this group.

We wanted to clarify a few points about the T5D phenotype, which is characterized by BMI < 18.5 kg/m^2^ [3]. Despite ongoing ambiguities regarding optimal diagnostic cut‐offs and whether anthropometric measurements are obtained at initial presentation or following metabolic stabilization, we recognize that exposure to persistent undernutrition remains one of the crucial characteristics of T5D. A detailed nutritional history of the study participants would help assess their nutritional status, although it can be challenging in resource‐limited areas. This challenge could be addressed using the Global Leadership Initiative on Malnutrition (GLIM) criteria [4], which combine simple, validated phenotypic and etiologic measures to identify malnutrition in adults. Additionally, the study participants were recruited from a young diabetes clinic at a tertiary referral centre in urban Dhaka. T5D is primarily reported in rural and socioeconomically disadvantaged populations [5, 6]. It may also be helpful to obtain data on other factors, such as intentional weight loss, genetic factors, or infections like HIV and TB, that may interact with insulin secretion and action, leading to lower BMI.

Thus, the cohort provides valuable characterization of lean, urban young‐onset non‐autoimmune diabetes. This is such an important diabetes phenotype that expanding catchment areas to include rural and socioeconomically deprived regions would also help identify individuals with this phenotype. Additionally, it would be of particular interest if future studies examining this condition correct glucotoxicity before analysing insulin sensitivity using HOMA‐IR. Given Bangladesh's burden of young‐onset diabetes, targeted recruitment of underweight participants with diabetes would be a substantial contribution, and this group appears well placed to undertake it.

## Author Contributions

**Wadivkar Pradnayshree:** conceptualization, writing – original draft, writing – review and editing, project administration, investigation. **Bahendeka Silver:** conceptualization, writing – review and editing, investigation. **Kibirige Davis:** conceptualization, investigation, writing – review and editing, supervision.

## Conflicts of Interest

The authors declare no conflicts of interest.

## Data Availability

Data sharing not applicable to this article as no datasets were generated or analysed during the current study.
